# 

**Published:** 1913-12

**Authors:** 


*THE BRISTOL MEDICO-CHIRURGICAL SOCIETY.*

*Editor:* P. WATSON-WILLIAMS, M.D.

WITH WHOM ARE ASSOCIATED

J. MICHELL CLARKE, M.A., M.D., LL.D.,

J. LACY FIRTH, M.S., M.D., JAMES SWAIN, M.S., M.D.

J. A. NIXON, B.A., M.B., *Assistant Editor.*

*Editorial Secretary:* J. M. FORTESCUE-BRICKDALE, M.A., M.D.

### CONTENTS.

*Page*

Specialism and the Medical Curriculum, mainly in reference to Otology, Rhinology and Laryngology. By P. Watson-Williams, M.D. Lond. ... ... ... ... ... ... ... ... ... 289

Protective Ferments. By I. Walker Hall, M.D. Vict. ... ... 304

On the Cerebral Symptoms of Lobar Pneumonia in Children. By F. H. Edgeworth, M.D., D.Sc. ... ... ... ... ... 308

Medical Observations in the Clifton Zoological Gardens, with Notes on the Birth of two Russian Bear Cubs in Captivity. By A. J. Harrison, M.B. Lond. ... ... ... ... ... ... 318

Splenectomy for Splenomegaly. By J. A. Nixon, M.B. Cantab., F.R.C.P. ... ... ... ... ... ... ... ... ... 325

Splenectomy in Banti's Disease. By E. W. Hey Groves, M.S. Lond., F.R.C.S. ... ... ... ... ... ... ... ... ... 331

Pericolitis. By T. Carwardine, M.S. Lond., F.R.C.S. ... ... ... 333

An Apparatus for the Intratracheal Administration of Ether. By F. E. Shipway, M.A., M.D. Cantab. ... ... ... ... ... 341

A Combined Manometer and Safety Valve for Intratracheal Anæsthesia. By Stuart V. Stock, M.B., B.S., F.R.C.S. and J. D. Fry, B Sc. ... ... ... ... ... ... ... ... ... ... 344

A Simple Apparatus for Intratracheal Anæsthesia. By E. W. Hey Groves, M.S. Lond., F.R C S. ... ... ... ... ... ... 347

Progress of the Medical Sciences:

Medicine. By J. M. Fortescue-Brickdale, M.A., M.D., B.Ch. Oxon. 350

Surgery. By C Ferrier Walters, F.R.C.S. ... ... ... ... 354

Otology and Rhinology. By P. Watson-Williams, M.D. Lond. ... 360

Reviews of Books ... ... ... ... ... ... ... ... 365

Obituary ... ... ... ... ... ... ... ... ... ... 370

Editorial Note. ... ... ... ... ... ... ... ... ... 371

Therapeutic Notes. By J. M. Fortescue-Brickdale, M.A., M.D , B.Ch. Oxon. ... ... ... ... ... ... ... ... 373

The Library of the Bristol Medico-Chirurgical Society ... ... 375

Meetings of Societies ... ... ... ... ... ... ... ... 380

Local Medical Notes ... ... ... ... ... ... ... ... 383

